# Tanshinone Inhibits NSCLC by Downregulating AURKA Through Let-7a-5p

**DOI:** 10.3389/fgene.2020.00838

**Published:** 2020-08-07

**Authors:** Xiaomin Liu, Heng Zou, Yiqi Zhao, Hang Chen, Tanglin Liu, Zong Wu, Chenghao Yang, Qian Li, Yanli Li

**Affiliations:** ^1^Lab for Noncoding RNA & Cancer, School of Life Sciences, Shanghai University, Shanghai, China; ^2^School of Life Sciences and Technology, China Pharmaceutical University, Nanjing, China

**Keywords:** NSCLC, tanshinones, let-7a-5p, AURKA, tumor suppressor

## Abstract

Lung cancer is the most deadly malignancy in the last decade, accounting for about 1.6 million deaths every year globally. Tanshinone is the constituent of *Salvia miltiorrhiza*; it has been found that they influence tumorigenesis. However, the role of tanshinones on lung cancer is still not clear. Let-7a-5p, a short non-coding RNA, is regarded as a suppressor gene in tumorigenesis. Herein, we verified that let-7a-5p is significantly downregulated in non-small-cell lung cancer (NSCLC) tissues and cell lines. Tanshinone suppressed the expression of aurora kinase A (AURKA), inhibited cell proliferation, and arrested cell cycle progression. Our results showed that tanshinones suppressed NSCLC by upregulating the expressions of let-7a-5p via directly targeting AURKA. Besides, the data reveal that the knockdown of AURKA can also inhibit cell proliferation, arrest cell cycle, and promote cell apoptosis. Furthermore, this study demonstrates that AURKA was negatively correlated with let-7a-5p in NSCLC patient tissues. Taken together, our findings suggest that tanshinone inhibits NSCLC by downregulating AURKA through let-7a-5p. Tanshinones and let-7a-5p have the potential to be candidates for drug development of NSCLC. In conclusion, this study revealed that tanshinones with miRNA linking lead to partial mechanism in NSCLC.

## Introduction

Lung cancer is the leading cause of cancer-related deaths worldwide, and nearly 80% of lung cancer cases are currently classified as non-small-cell lung cancer (NSCLC) (Torre et al., 2015). The 5-year survival rate remains very low due to disease recurrence or metastasis, despite great advances having been made in the treatment of NSCLC (Fassina et al., 2011). The prevalence and lethality of this disease highlight the importance of investigating the mechanisms involved in the tumorigenesis of NSCLC, as well as prognosticating potential therapeutic targets for its treatment. Current treatment is a combination of intensive multi-agent chemotherapy and surgery (Osmani et al., 2018). However, high-dose chemotherapy has many adverse effects. *Salvia miltiorrhiza* Bunge is one of the most commonly used traditional Chinese medicines in clinical practice (MEIm et al., 2019). Its traditional functions are activating blood circulation, removing blood stasis, relieving pain, and nourishing blood (Chen and Chen, 2017; Li F.et al., 2019; Li et al., 2019a). In China, the *S. miltiorrhiza* compound is mainly used for the prevention and treatment of coronary heart disease and angina (Chen and Chen, 2017; MEIm et al., 2019). In the United States, the *S. miltiorrhiza* compound has reached phase III of clinical trials and is mainly used for the treatment of chronic angina (Ren et al., 2019). The main effective components of *S. miltiorrhiza* are tanshinone ether and ethanol extracts taken from the roots; the most abundant content of tanshinone is tanshinone I (T1), tanshinone type IIA (T2A), and implicit tanshinone (cryptotanshinone, CT) (Zhou et al., 2005; Li et al., 2019a). In recent years, research has shown that tanshinone has a cytotoxic effect on tumor cells, mainly through the induction of apoptosis, inhibiting angiogenesis, cell cycle progression, cell invasion, and metastasis (Li F.et al., 2019; Lin et al., 2019; Liu et al., 2019).

MicroRNAs (miRNAs) are a class of small non-coding RNA molecules (19–22 nucleotides), which play key roles in regulating protein expression through inhibiting translation or inducing mRNA degradation by binding to the 3′-untranslated region (3′-UTR) of target mRNAs (Bartel, 2009). More and more evidence show that miRNAs play important roles in regulating cancer initiation and development (Calin and Croce, 2006; Moles, 2017). Besides, miRNAs are critical for tumorigenesis and have been characterized as oncogenes or tumor suppressors (Li et al., 2017; Xiao et al., 2019; Yasukawa et al., 2019; Zhang et al., 2019; Weidle et al., 2020). Yet the role of let-7a-5p in NSCLC has not been completely investigated.

In the present study, we found that let-7a-5p was significantly downregulated in NSCLC tissues and cell lines. The results elucidated the mechanism by which tanshinone inhibits NSCLC by downregulating aurora kinase A (AURKA) through let-7a-5p. Our results indicated that tanshinones and let-7a-5p have the potential to be positive candidates in the drug development of NSCLC.

## Results

### AURKA Is Upregulated in NSCLC

**FIGURE 1:**
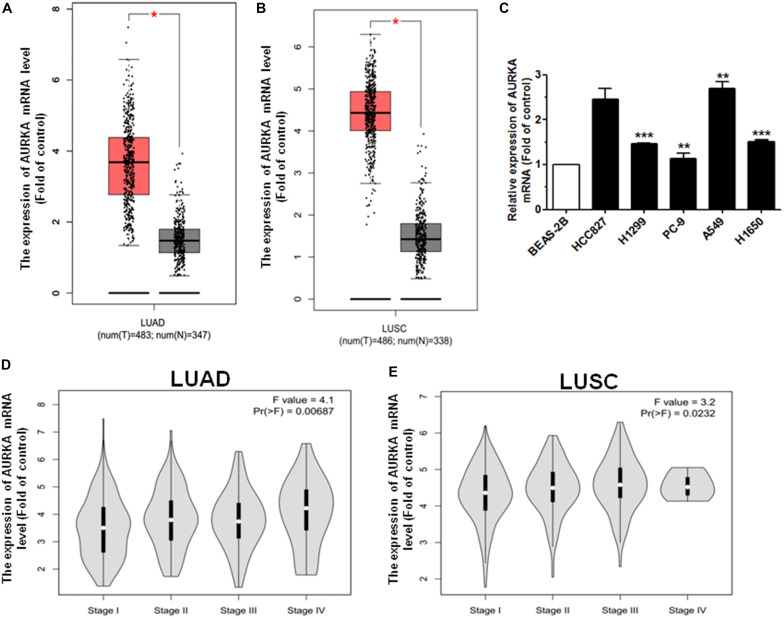
AURKA is upregulated in NSCLC. **(A)** The expression mRNA level of AURKA is upregulated in LUAD samples with 483 tumor (T) samples compared with 347 normal (N) samples, using the GEPIA online database. **(B)** The expression mRNA level of AURKA is upregulated in LUSC samples with 486 tumor (T) samples compared with 338 normal (N) samples, using the GEPIA online database. **(C)** The expression mRNA level of AURKA in five human NSCLC cell lines. BEAS-2B cells were used for the normal control comparison. **(D,E)** Expression of AURKA as related to pathological stage in LUAD and LUSC cases (ANOVA for statistics). *P < 0.05, **P < 0.01, and ***P < 0.001.

In order to analyze the effect of AURKA expression in NSCLC patients, we generated the level of AURKA expression using the GEPIA online database^1^. Among the lung adenocarcinoma (LUAD) and lung squamous cell carcinoma (LUSC) cases, we found that when compared with non-tumor (N) tissues, the level of AURKA expression was upregulated in tumor (T) tissues (Figures 1A,B). Next, we analyzed the AURKA expression level in NSCLC cell lines. As a result, AURKA was significantly upregulated in five NSCLC cell lines compared with the normal bronchial epithelial cells (BEAS-2B) (Figure 1C). Additionally, the upregulation of AURKA was related to the pathological stage, the expression levels of AURKA increased as the cancer stages increased (Figures 1D,E). However, the online database has no demographic information such as race, gender, body weight, age, and sample size. We will continue to analyze clinical tissue samples with more demographic information. These results suggested that high levels of AURKA may act as a tumor oncogene in NSCLC.

### AURKA Is Downregulated by T1 and T2A in NSCLC Cells

**FIGURE 2:**
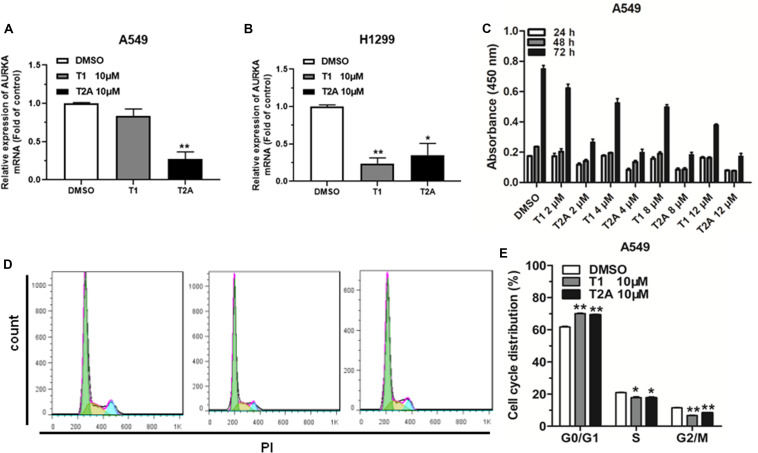
AURKA is downregulated by T1 and T2A in NSCLC cell, and T1 and T2A can inhibit cell proliferation and arrest cell cycle progression. **(A,B)** The mRNA levels of AURKA were detected by qRT-PCR in A549 and H1299 cell treatment with T1/T2A for 48 h. **(C)** The proliferation of A549 cell line, as measured by CCK-8 assay, following treatment with T1 and T2A, under the drug concentration gradient. **(D,E)** The cell cycle distributions of A549 cell treatment with T1/T2A were detected by flow cytometry. *P < 0.05 and **P < 0.01.

Based on the results of our previous studies, we found that tanshinone has an anticancer effect on NSCLC. Quantitative real-time PCR (qRT-PCR) was used to analyze the expression levels of AURKA in NSCLC cells treated with 10 μM T1 and T2A for 48 h. The results revealed that the relative expression levels of AURKA significantly decreased after treatment with T1 and T2A in A549 and H1299 cell lines (Figures 2A,B). To investigate the role of T1 and T2A in cell proliferation and cell cycle of NSCLC cells, proliferation of NSCLC cells was assessed, and the drug concentration gradient was set to determine the optimal concentration by a cell counting kit-8 (CCK-8) assay (Figure 2C). Results indicated that T1 and T2A exhibited a significant decrease in cell proliferation. Flow cytometry analysis indicated that T1 and T2A were involved in the cell cycle progression, which led to a decrease in the rate of cell proliferation (Figures 2D,E).

### Knockdown of AURKA Suppresses the Proliferation and Arrests Cell Cycle in NSCLC

**FIGURE 3:**
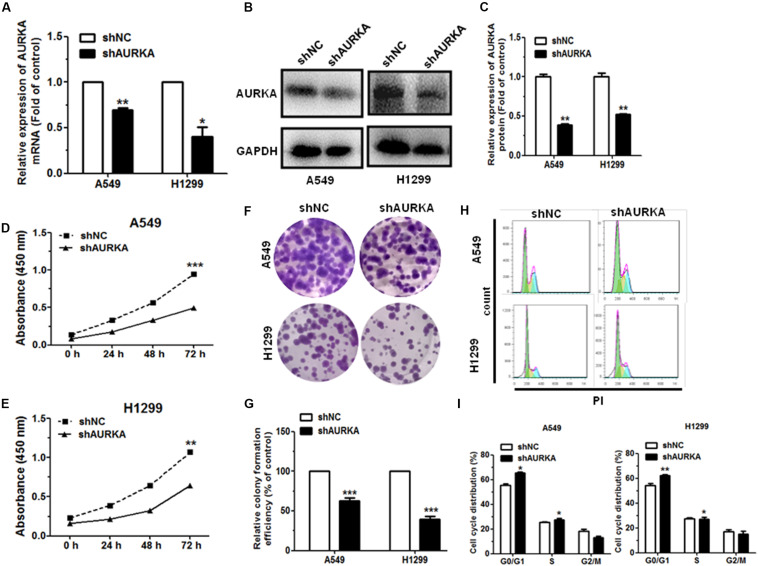
Downregulation of AURKA can inhibit cell proliferation and impede cell cycle progression in NSCLC. **(A–C)** The levels of AURKA mRNA and protein level in A549 and H1299 cells transfected with AURKA shRNA (shAURKA) were measured by qRT-PCR and western blot, respectively. **(D,E)** Proliferation of A549 and H1299 cells with or without AURKA silencing. **(F,G)** Colony formation of A549 and H1299 cells with or without AURKA silencing. **(H,I)** The cell cycle distributions of A549 and H1299 cells with or without AURKA silencing were detected by flow cytometry. *P < 0.05, **P < 0.01, and ***P < 0.001.

To investigate the function of AURKA in NSCLC, we silenced AURKA through constructing short hairpin RNA (shRNA—shAURKA). Our results illustrated that the expression of AURKA mRNA and protein level were significantly downregulated in NSCLC cells transfected with shAURKA compared with the shNC group (Figures 3A–C). The growth curves showed that compared with the cells transfected with shNC, downregulation of AURKA suppressed cell proliferation in A549 and H1299 cells by using the CCK-8 assay (Figures 3D,E). We also performed colony formation assay to investigate cell proliferation function. Results showed that knockdown of AURKA could significantly inhibit colony formation in A549 and H1299 when compared with the shNC group (Figures 3F,G). Furthermore, we investigated the effect of AURKA on cell cycle and cell apoptosis. Flow cytometry analysis revealed that downregulation of AURKA dramatically suppressed cell cycle progression and promoted cell apoptosis in A549 and H1299 cells than that in shNC group (Figures 3H,I and Supplementary Figures 1A,B).

### Let-7a-5p Directly Targets AURKA Which Is Potential for Drug Development

**FIGURE 4:**
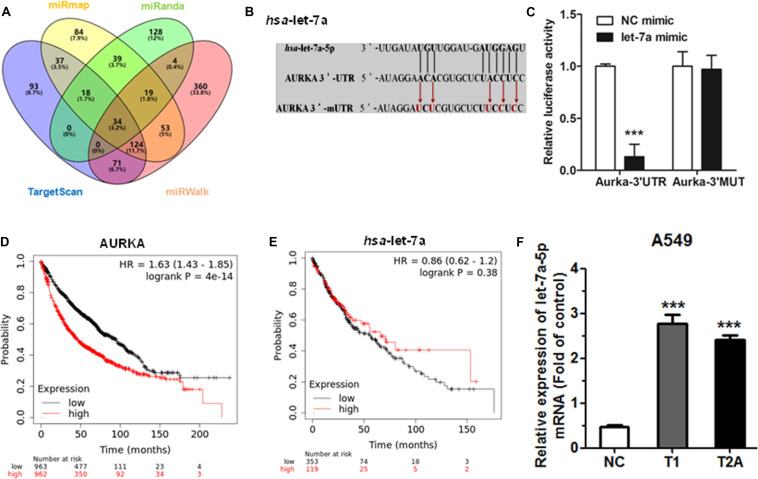
Let-7a-5p directly targets AURKA. **(A)** TargetScan, miRanda, miRWalk, and miRmap were used to predict direct target miRNAs of AURKA. **(B)** AURKA WT 3′-UTR contains a predicted let-7a-5p binding site. The data show alignment of let-7a-5p with AURKA WT 3′-UTR, and arrows indicate mutagenesis nucleotides. **(C)** Dual luciferase assay in HEK-293T cells co-transfected with WT or mut AURKA 3′-UTR luciferase vectors and let-7a-5p mimic or NC mimic. **(D)** The effect of AURKA expression mRNA levels on the overall survival of 1,925 NSCLC patients (cutoff value: 657, *P*-value: 4.0e–14). From Kaplan–Meier curve for Kaplan–Meier plotter datasets. **(E)** The effect of let-7a-5p expression mRNA levels on the overall survival of 472 NSCLC patients (cutoff value: 8,940, *P*-value: 0.384). From Kaplan–Meier curve for Kaplan–Meier plotter datasets. **(F)** The mRNA levels of let-7a-5p were detected by qRT-PCR after treatment with T1/T2A 48 h in A549 cells. ***P < 0.001.

In order to predict the possibility of miRNA targeting AURKA, we used the bioinformatics tool TargetScan^2^, miRanda^3^), miRWalk^4^, and miRmap^5^ to screen the putative miRNA. We found that AURKA is the potential target for let-7a-5p (Figure 4A). The mutant and wild-type binding sites of let-7a-5p to AURKA are shown in Figure 4B. Subsequently, in order to determine whether or not AURKA is a direct target of let-7a-5p, AURKA wild-type 3′-UTR (AURKA WT 3′-UTR) was cloned into the pGL3 vector (pGL3-AURKA WT 3′-UTR), downstream of the luciferase open reading frame (ORF). In addition, the pGL3 vector with the mutation of AURKA 3′-UTR (pGL3-AURKA mut 3′-UTR) was recombinant to validate target specificity, using a QuikChange Mutagenesis kit to construct. There was a distinct decrease in the relative luciferase activity of the reporter gene cotransfected with AURKA 3′-UTR (pGL3-AURKA WT 3′-UTR), pRL vector, and let-7a-5p mimic compared to the control (cotransfected with AURKA 3′-UTR, pRL vector, and NC mimic). Inversely, cotransfection of let-7a-5p with AURKA 3′-mUTR (pGL3-AURKA mut 3′-UTR) resulted in no significant change in luciferase activity, supporting miRNA/target 3′-UTR specificity (Figure 4C).

Next, we generated a Kaplan–Meier survival curve of NSCLC patients with low or high AURKA expression using the Kaplan–Meier plotter online database^6^ (Figure 4D). Among the 1,926 sample cases, we found that NSCLC patients with high AURKA expression had lower survival rates. Inversely, NSCLC patients with low let-7a-5p expression had lower survival rates (Figure 4E). However, the limitation of the Kaplan–Meier plotter online database is having no demographic information. We will continue to mine for clinical tissue sample data. To further investigate the effect of let-7a-5p after T1 and T2A treatment, qRT-PCR was performed to compare the relative miRNA expression. As a result, let-7a-5p showed the most significant increase in expression levels compared with the control group after T1 and T2A treatment for 48 h (Figure 4F). Therefore, T1 and T2A can influence the expression of let-7a-5p.

## Discussion

Lung cancer is an aggressive malignant tumor and grows from the cancerous cells of mesenchymal origin, which is driven by the sequential accumulation of genetic and epigenetic changes in oncogenes and tumor suppressor genes, especially in NSCLC (Atkinson et al., 2019; Lahouel et al., 2019). Thus, it is of great importance to identify novel and specific molecular markers for NSCLC diagnosis and treatment. In this study, there are four points in our findings. Firstly, AURKA expression was downregulated in tissues or cell lines. Secondly, we further validated that treatment with T1 and T2A inhibited cell proliferation and arrested cell cycle progression, in which AURKA was downregulated in NSCLC cells. Thirdly, downregulation of AURKA suppressed the proliferation and arrested cell cycle in NSCLC. Finally, the luciferase reporter assay confirmed that let-7a-5p directly targets AURKA, and the expression level of let-7a-5p was significantly upregulated by T1/T2A. These results revealed that under the tanshinone treatment, the AURKA direct regulator let-7a-5p was a potential tumor suppressor miRNA for NSCLC.

MicroRNAs plays an important role in tumorigenesis by regulating the cell cycle, cell differentiation, and cell proliferation (Xu et al., 2019; Yang et al., 2019). Our previous studies have revealed that tanshinones could inhibit NSCLC by suppressing AURKA via upregulating the expressions of miR-32 (Ma et al., 2015). Moreover, CT could suppress expression of EGFR in NSCLC cells. CT and miR-146a-5p have the potential to be positive candidates in drug development of NSCLC (Qi et al., 2019).

**FIGURE 5:**
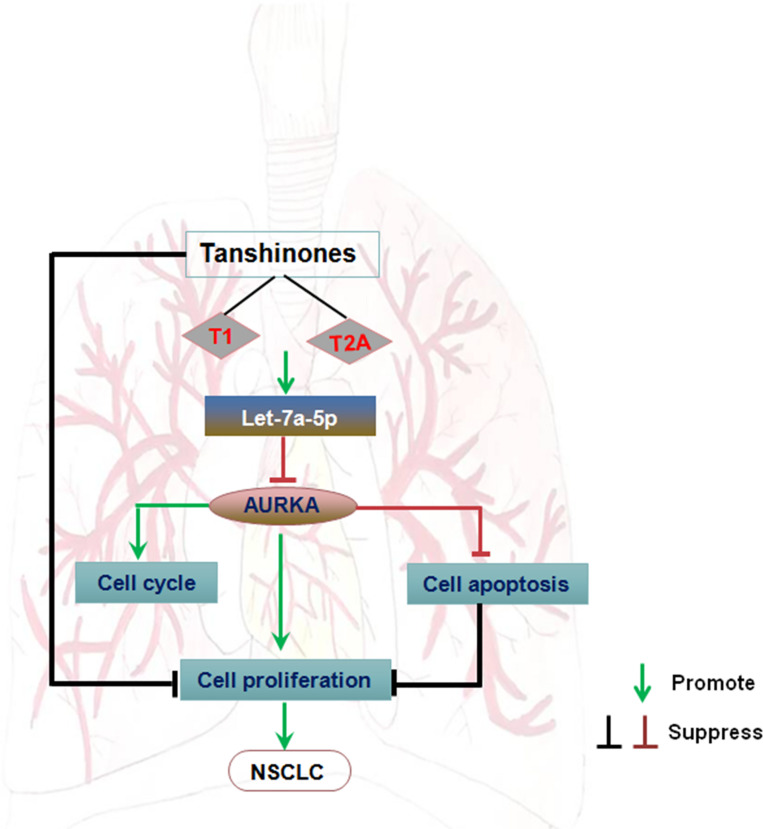
The regulatory network of the tanshinones/AURKA/let-7a-5p axis in NSCLC carcinogenesis. Arrows indicate promotion, and T-shaped arrows indicate suppression.

In this research, we find the let-7a-5p is a tumor-suppressive miRNA. It has been demonstrated that let-7a-5p is downregulated in diverse malignancies, such as colorectal cancer (Liu et al., 2016), breast cancer (Chen et al., 2019), lung cancer (Li et al., 2018), and gastrointestinal cancers (Konno et al., 2019). Additionally, let-7a-5p plays its tumor-suppressive roles in inhibiting cell proliferation and migration and in promoting apoptosis through regulating different oncogenes. Bai et al. (2019) indicated that let-7a-5p regulates cell proliferation, migration, and the doxorubicin resistance of prostate cancer through targeting EGFR. Liu et al. (2018) revealed that the lncRNA NEAT1/let-7a-5p axis regulates the cisplatin resistance by targeting Rsf-1 in nasopharyngeal carcinoma and modulating cell proliferation. Liu et al. (2016) found that downregulation of let-7a-5p can predict lymph node metastasis and prognosis in colorectal cancer. We also found that with the increase of tanshinone (T1 and T2A) concentration, there is a better effect of inhibiting lung cancer *in vitro* (Ma et al., 2015). Here, we demonstrated that tanshinone functionally participated in suppressing cell proliferation, arresting cell cycle, and promoting cell apoptosis by downregulating AURKA through let-7a-5p (Figure 5).

At the molecular level, our data showed that AURKA could be a direct target of let-7a-5p. The Aurora kinase family is composed of three serine/threonine kinases, Aurora A, Aurora B, and Aurora C. Among these, Aurora A and Aurora B play central roles in mitosis, whereas Aurora C acts unique roles in meiosis. AURKA, which is a serine–threonine kinase encoded by the AURKA gene, is responsible for regulating mitotic processes in mammalian cells, including chromosome segregation, spindle assembly, and centrosome maturation (Fu et al., 2007). Another member of the Aurora kinase subfamily of conserved serine/threonine kinases—AURKB—starts at early G2 phase and localizes to the chromosomes in the prophase, the centromere in prometaphase and metaphase, the central spindle in anaphase, and the mid-body in cytokinesis (Sugimoto et al., 2002). AURKA plays a critical role in chromosomal instability, cell cycle progression, and maturation (Marumoto et al., 2005; Liao et al., 2018). Recent investigation found that AURKA was involved in a variety of human cancers. For example, Chen et al. (2017) suggested that AURKA was reactivated in metastasis of irradiated hepatocellular carcinoma (HCC) through facilitating epithelial–mesenchymal transitions (EMTs) and cancer stem cell (CSC) properties. Soleymani Fard et al. (2019) showed that upregulation of AURKA might promote gastric cancer progression. de Jong et al. (2019) indicated that a high expression of AURKA correlated with cell cycle progression during chondrosarcoma cell survival. Our findings were consistent with previous studies that found AURKA to be upregulated in NSCLC and to promote cell proliferation.

## Conclusion

In conclusion, our study shows that tanshinone inhibits NSCLC by downregulating AURKA through let-7a-5p. Therefore, tanshinones and let-7a-5p have the potential to be positive candidates in the drug development of NSCLC.

## Materials and Methods

### Cell Culture

H1299, H1650, and HCC827 cells were cultured in RPMI-1640 medium (Gibco, Gaithersburg, MD, United States) with 10% fetal bovine serum (FBS, HyClone Laboratories, Logan, UT, Untied States). In addition, A549, HEK-293T, and PC-9 cells were cultivated in Dulbecco’s modified Eagle’s medium (DMEM, Gibco, Gaithersburg, MD, United States) with 10% FBS. All media were supplemented with 100 U/ml penicillin and 100 μg/ml streptomycin (HyClone, Logan, UT, United States). All the cells were cultured in the condition of v/v 5% CO_2_ at 37°C in a humidified cell incubator. H1299, PC-9, H1650, and A549 cell lines were obtained from the American Type Culture Collection (Manassas, VA, United States). HCC827, BEAS-2B, and HEK-293T cell lines were gained from the China Academy of Sciences (Shanghai, China).

### RNA Extraction and qRT-PCR

After the indicated treatment with T1 and T2A (10 μM), total RNA was extracted with the TRIzol reagent (Sangon Biotech) following the manufacturer’s standards. The cDNA reversed by the miRNA was used as one of the substrates in qRT-PCR. The miRNA cDNA library was established by a SuperMixQuantiMir cDNA kit (TransGen Biotech, Beijing, China) using a M-MLV RTase cDNA synthesis kit (TaKaRa, Dalian, China). The measurement of expression levels was shown by a SYBR Green PCR mix (Takara). miRNAs were normalized to U6 snRNA as internal control, and mRNAs were 18S RNA as internal control. Relative quantification (2^–Δ^ ^Δ^ ^CT^) was the method used to analyze the results. For miRNA expression analysis, the miR-specific forward primers and the universal reverse primers were used. The let-7a-5p-specific forward primer sequence was 5′-TGAGGTAGTAGGTTGTATAGTT-3′. The forward primer sequence of reference U6 snRNA was 5′-CTCGCTTCGGCAGCACA-3′, and the reverse primer was 5′-AACGCTTCACGAATTTGCGT-3′. The forward primer sequence for AURKA mRNA was 5′-CATCTTCCAGGAGGA CCACT-3′, and the reverse primer was 5′-CAAAGAA CTCCAAGGCTCCA-3′. The 18S RNA forward primer was 5′-AGGAATTCCCAGTAAGTGCG-3′, and the reverse primer was 5′-GCCTCACTAAACCATCCAA-3′. The AURKA 3′-UTR forward primer was 5′-GCTCTAGAGCATAAGG ATGATGCGAGGG-3′, and the reverse primer was 5′-CGGAATTCCGTGGAGGATGAAGTGGAGA-3′. The AURKA 3′-mUTR forward primer was 5′-AATAACCCTGAAAAA TAATAATTGAATTCCTTTTCTA-3′, and the reverse primer was 5′-ATGCTAGAAAAGGAATTCAATTATTA TTTTTCA GGGT-3′. The shAURKA forward primer was 5′-CCG GGGTCTTGTGTCCTTCAAATTCCTCGAGGAATTTGAAGG ACACAAGACCTTTTTG-3′, and the reverse primer was 5′-AA TTCAAAAAGGTCTTGTGTCCTTCAAATTC CTCGAGGAATTTGAAGGACACAAGACC-3′.

### Cell Transfection

At 80% of HEK-293T cell confluence, cells were transfected transiently with 100 nM of a chemically synthesized negative control mimic (NC) or let-7a-5p mimic, which was purchased from the RiboBio Company (Guangzhou, China), by using Lipofectamine 2000 (Thermo Fisher Scientific, Boston, MA, United States) according to the manufacturer’s recommendations. After transfection at 24–48 h, subsequent experiments were performed in treated cells, such as cell cycle analysis and qRT-PCR.

### Cell Proliferation Assay

Cell proliferation was measured according to the CCK-8 assay (Dojindo, Japan) every 24 h for three times. Briefly, for cellular drug treatment, 2 × 10^3^ cells were plated in every 96-well plate, and different concentrations of T1 and T2A (2, 4, 8, and 12 μM) were added to the wells for 24, 48, 72 h, and cells were incubated in a 5% CO_2_ humidified cell incubator with 37°C. Cells were turned to culturing for 2.5 h after adding CCK-8. The light absorption rate at 450 nm was measured with a microplate reader daily to indicate the cell proliferation rate. This experiment was carried out independently at least thrice.

### Cell Cycle Analysis

After the indicated treatment with T1 and T2A (10 μM), 1 × 10^5^ treated cells were obtained and were stored in absolute 70% ethanol at −20°C overnight. Cells were precipitated with 20 μl FBS, added with PBS to wash it after suspending, then added with RNase A (100 ng/mL) to resuspend for 30 min at 37°C, and dyed with propidium iodide (PI) (50 ng/ml) at room temperature in the dark for 15 min. Cells were filtered with a 200-mesh nylon membrane and detected with A MoFlo XDP flow cytometer (Beckman Coulter, United States). FlowJo software was used to analyze the data. Each experiment was repeated at least three times independently.

### Cell Apoptosis Analysis

Usually, the annexin V–fluorescein isothiocyanate apoptosis detection kit (BD Pharmingen, San Diego, CA, United States) is the main method used to determine cell apoptosis level according to the manufacturer’s instruction. H1299 and A549 cells were resuspended with a mix of annexin V–fluorescein isothiocyanate and PI in 1 × binding buffer solution and then incubated for 15 min at ambient temperatures in the darkness. Apoptotic cells were analyzed using a MoFlo XDP flow cytometer (Beckman Coulter, Inc., Brea, CA, United States). Experiments with triplicates should be performed at least thrice separately.

### Protein Extraction and Western Blot Analysis

The total protein was extracted by RIPA cell lysis buffer (CWBIO, Beijing, China), and this extract can be used in the next experiment (western blot) after determining its concentration. The total protein was quantified through a protein BCA assay kit (Bio-Rad, Hercules, CA, United States). Western blotting was based on the antigen–antibody reaction and separated by SDS–polyacrylamide gel electrophoresis. Then the gel should be transferred to a polyvinylidene difluoride membrane (Millipore Corporation, Billerica, MA, United States). After blocking with 5% milk made from skim milk powder for 1 h, the membrane should be incubated with rabbit anti-AURKA and anti-GAPDH antibodies in the refrigerator (4°C) overnight and should be washed three times and incubated with goat-anti-rabbit secondary antibody coupling horseradish peroxidase (1:1,000, Cell Signaling Technology). The expression levels of protein were mathematically equivalent to the intensity of fluorescence signals, which are detected by a chemiluminescent horseradish peroxidase substrate (Millipore), and images were obtained with an E-Gel Imager (Bio-Rad, Hercules, CA, United States).

### Luciferase Reporter Assay

Recombinant expression vectors were confirmed by sequencing (Sangon Biotech, Shanghai, China). 3′-UTR luciferase recombinant plasmid was presented as follows: the predicted binding site of let-7a-5p was cloning behind the firefly luciferase in the pGL3 vector (Promega, Madison, WI, United States). Meanwhile, the AURKA-3′-mUTR plasmid with the mutated binding site was cloned at the same site, and there was site-directed mutagenesis by quick-change PCR using mutated primer pairs and Pfu polymerase (Takara). TargetScan, miRanda, miRWalk, and miRmap were all used to predict the target genes of let-7a-5p. The luciferase vector AURKA-3′-UTR or AURKA-3′-mUTR (final concentration of 100 nM for let-7a-5p mimic or NC mimic) was transiently cotransfected with 20 ng of plasmid expressing the renilla luciferase gene (pRL, Promega) into HEK-293T cells (cultured in 24-well plate). After 48 h of transfection, the luciferase activity was measured by Orion II Microplate Illuminometer (Titertek-Berthold, South San Francisco, CA, United States). Relative activities were shown as the fold change under normalization to renilla luciferase activity. Experiments with triplicates should be performed at least thrice separately.

### Statistical Analysis

Results are expressed as the group means ± SEM and analyzed using GraphPad Prism 8 software, using *t*-tests for two-group comparisons and ANOVA for more than two-group comparisons. Differences were considered statistically significant when *P* < 0.05.

## Data Availability Statement

The raw data supporting the conclusions of this article will be made available by the authors, without undue reservation, to any qualified researcher.

## Author Contributions

YL and QL designed the study and approved the final version of the submitted manuscript. XL and HZ developed the methodology and researched the data. XL, HZ, YZ, HC, TL, ZW, and CY performed the experiments. XL, HZ, and YZ analyzed and interpreted the data. YL, XL, HZ, and YZ wrote and edited the manuscript. All authors read and approved the contents of the manuscript and its publication.

## Conflict of Interest

The authors declare that the research was conducted in the absence of any commercial or financial relationships that could be construed as a potential conflict of interest.

## Footnotes

1 http://gepia.cancer-pku.cn/detail.php?gene=AURKA

2 http://www.targetscan.org/vert_72/

3 http://www.microrna.org/microrna/home.do

4 http://mirwalk.umm.uni-heidelberg.de/

5 https://mirmap.ezlab.org/

6 www.kmplot.com/analysis

## Supplementary Material

FIGURE S1 Downregulation of AURKA can promote cell apoptosis in NSCLC. **(A,B)** The rate of apoptosis was analyzed by flow cytometry following transfected with or without AURKA silencing. **P* < 0.05, ***P* < 0.01.
