# Supplementary figures and images for: Tanshinone Inhibits NSCLC by Downregulating AURKA Through Let-7a-5p
Source: Front Genet. 2020 Aug 7;11:838. doi: 10.3389/fgene.2020.00838 (PMC7427477; doi:10.3389/fgene.2020.00838)

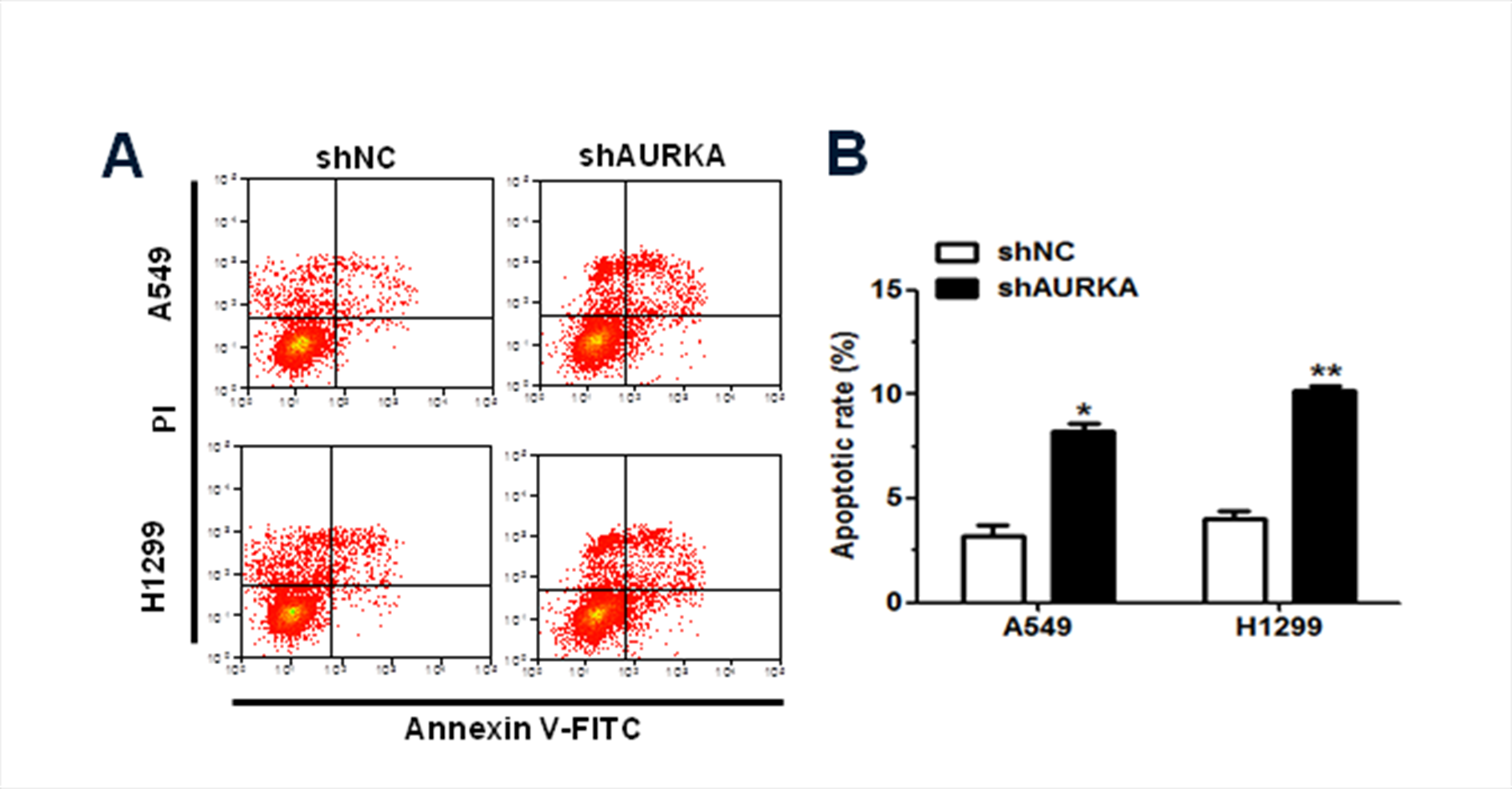

Supplement: FIGURE S1 — Downregulation of AURKA can promote cell apoptosis in NSCLC. (A,B) The rate of apoptosis was analyzed by flow cytometry following transfected with or without AURKA silencing. *P < 0.05, **P < 0.01. [file Image_1.tif]
